# Low plasma edoxaban concentrations during therapeutic drug monitoring: a single-center retrospective study

**DOI:** 10.1007/s00210-026-05486-4

**Published:** 2026-06-03

**Authors:** Paulina Flis, Roza Chaireti, Rickard E. Malmström, Karolina Nowinski

**Affiliations:** 1 Department of Clinical Pharmacology, Karolinska University Hospital, Stockholm, Sweden; 2 Department of Laboratory Medicine, Karolinska Institutet, Stockholm, Sweden; 3 Department of Hematology, Karolinska University Hospital, Stockholm, Sweden; 4 Department of Medicine Solna, Karolinska Institutet, Stockholm, Sweden; 5 Department of Molecular Medicine and Surgery, Karolinska Institutet, Stockholm, Sweden; 6 Clinical Pharmacology C1:68, Karolinska University Hospital, 141 86 Huddinge, Sweden

**Keywords:** Edoxaban, Direct oral anticoagulant, Therapeutic drug monitoring, Pharmacokinetics, Renal function, Glomerular filtration rate

## Abstract

In our therapeutic drug monitoring (TDM) laboratory, over one-third of approximately 350 analyzed samples from patients on edoxaban were < 12 ng/mL, which is below concentrations reported in clinical trials. Our study aimed to explore the effect of factors potentially associated with low edoxaban concentrations in patients monitored with TDM. A retrospective cohort study of previously analyzed edoxaban plasma concentrations collected at Karolinska University Hospital between November 2017 and May 2023 was performed. Low concentration was defined as < 12 ng/mL (30 mg) and < 16 ng/mL (60 mg). Fifty samples from 34 individuals were included. No significant difference in trough concentration between 30 and 60 mg dose group was found. Low concentrations were observed in 42% of samples. Higher estimated glomerular filtration rate (eGFR) was found among patients with low edoxaban concentrations (*p* = 0.004); however, the correlation between eGFR and concentration was weak (rho =  − 0.4; *p* < 0.05). Off-label dose reduction (*n* = 4) and drug-drug interactions (DDIs) (*n* = 4) were found in cases with low concentrations. In a TDM-setting, low trough concentrations of edoxaban were frequently observed. Potential explanations include normal renal function, drug-drug interactions, and off-label dose reductions. These findings suggest that TDM may be beneficial in selected clinical scenarios. However, the study is limited by its small sample size and selected study population.

**Supplementary Information:**

The online version contains supplementary material available at 10.1007/s00210-026-05486-4.

## Introduction

### Background

Direct oral anticoagulants (DOACs), including apixaban, dabigatran, edoxaban, and rivaroxaban, are commonly prescribed for stroke prophylaxis in patients with atrial fibrillation (AF) and for the prevention and treatment of venous thromboembolism (VTE) (Kirchhof et al. 2024; Stevens et al. 2024). In Stockholm County, approximately 1200 individuals were treated with edoxaban in year 2023, which is lower than the number of patients treated with the other approved DOACs, but the prevalence of edoxaban treatment in Stockholm is continuously increasing since its introduction in 2016 (“The Swedish prescribed drug register” n.d.).

The recommended dose of edoxaban is 60 mg administered once daily and should be decreased to 30 mg once daily in patients with moderate or severe renal impairment, low body weight (≤ 60 kg), or concomitant use of the P-glycoprotein (P-gp) inhibitors ciclosporin, dronedarone, erythromycin, or ketoconazole (“SmPC edoxaban” n.d.). Edoxaban pharmacokinetics is characterized by a high correlation between the plasma trough concentration and the efficacy and safety of treatment (Ruff et al. 2015). Monitoring of plasma concentrations of DOAC (therapeutic drug monitoring, TDM) is not generally required even though this can be warranted in certain situations such as bleeding, renal impairment, drug-drug interactions (DDI), or suspicion of treatment failure (thromboembolic events) (Steffel et al. 2021a).

A direct method for measuring the plasma concentration of edoxaban has been developed at the Department of Clinical Pharmacology, Karolinska University Hospital, Stockholm, Sweden, and has been available in clinical practice since 2017. The method quantifies the parent drug, but not the active metabolites. At the TDM laboratory, we observed that the concentration of more than one third of the approximately 350 analyzed samples were below 12 ng/mL which is below the 5th percentile of concentrations measured in the ENGAGE AF-TIMI trial (Steffel et al. 2021b). Based on this finding, we initiated a study to explore factors potentially affecting edoxaban concentrations in a clinical cohort of patients treated at the Karolinska University Hospital. The main hypothesis was that renal function is the main determinant of low edoxaban concentration. The overall aim of this study was to optimize efficacy and safety of treatment with edoxaban.

### Objectives

The objectives were to identify the proportion of patients with low edoxaban concentration and off-label dose reductions in a clinical cohort and to analyze the correlation between concentration and covariates such as renal function, weight, age, and drug-drug interactions.

## Materials and methods

### Study design

This was a retrospective analytical cohort study based on the results of previously analyzed samples of edoxaban concentrations in plasma. The study was approved by the Swedish Ethical Review Authority.

### Setting

The study was conducted at the Department of Clinical Pharmacology, Karolinska University Hospital, Stockholm, Sweden. All samples were analyzed at the TDM laboratory at the Department of Clinical Pharmacology at Karolinska University Hospital between November 8, 2017 (start date of the method), and May 26, 2023. The method of edoxaban measurement in plasma used at our laboratory is a validated routine method and the method is part of an external quality control programme (External quality Control of diagnostic Assays and Tests (ECAT) Foundation) since 2019. The laboratory prepares their own internal quality control samples from certified reference material. Analysis is performed through reversed-phase high-pressure liquid chromatography with tandem mass spectrometry (HPLC–MS/MS, spectrometer: Xevo TQ-S micro, Waters). The current method analyzes apixaban, dabigatran, edoxaban, and rivaroxaban. The method is validated for edoxaban concentrations > 2 ng/mL.

### Study participants (patients)

The study population consisted of both outpatients and inpatients at the Karolinska University Hospital whose blood samples were sent to the Clinical Pharmacology laboratory for measurement of plasma concentration of edoxaban. Only trough values at steady state, defined as samples obtained 15–30 h after last dose following at least 3 days of treatment and with an estimated glomerular filtration rate (eGFR) registered, were included in the analysis. For analysis of drug-drug interactions and treatment indication, each patient was included only once.

Each patient could contribute with several separate samples of edoxaban concentrations with a corresponding eGFR, weight, age, and dose for each sample. For analysis of edoxaban concentrations, each sample was included in the analysis.

To account for within-patient clustering, a sensitivity analysis of the above comparisons was performed at the patient level using median concentration per individual. One patient was treated with both 30 mg and 60 mg and was therefore excluded from the patient-level analysis.

For the two patients receiving dosages of both 15 mg and 30 mg over consecutive time, only the median concentration during the 30 mg dosing period was included in the patient-level analysis.

### Variables and definitions

The following variables were included in the analyses: age, sex, weight (kg), concentration of edoxaban (ng/mL) and corresponding timepoint of obtained sample, time of last dose, eGFR, concomitant medication with pharmacokinetic interaction potential, and indication for treatment with edoxaban.

Calculation of eGFR was based on creatinine and calculated by the Lund-Malmö revised formula (LM rev) (Björk et al. 2020). Concomitant prescription of drugs with potential for a pharmacokinetic interaction within 1 month before blood sampling was included (Table S1). The drugs were chosen according to clinically relevant pharmacokinetic interactions in the European Heart Rhythm Association (EHRA) guidelines and the Swedish clinical decision support system Janusmed Interactions and Risk Profile. Interactions with edoxaban included those classified as “caution required due to risk of bleeding,” “consider avoiding,” or “contraindicated” according to EHRA-guidelines or classified as B-D interaction with edoxaban in Janusmed Interactions (Steffel et al. 2021a, b; Janusinfo n.d.). The presence of a registered ICD-10 diagnosis of AF at any timepoint before sampling and/or VTE within 1 year before sampling was included to determine the plausible indication for treatment (Table S2).

Off-label dosing was defined as a dose reduction from 60 to 30 mg once daily if none of the criteria for dose reduction according to the Summary of Product Characteristics (SmPC) was fulfilled (“SmPC edoxaban” n.d.).

Low edoxaban concentration was defined as < 12 ng/mL in patients treated with 30 mg and < 16 ng/mL in patients with 60 mg, respectively. High concentration was defined as > 29 ng/mL and > 43 ng/mL in the groups treated with 30 mg daily and 60 mg daily, respectively, based on the 95th percentile in ENGAGE AF-TIMI 48 trial (Steffel et al. 2021b). This was also in accordance with the European practical guide on the use of DOAC where expected plasma levels included edoxaban trough levels of 12–43 ng/mL (Steffel et al. 2021a). A sensitivity analysis was performed using thresholds from the HOKUSAI-VTE trial (edoxaban for venous thromboembolism). Low concentration was in this analysis defined as < 8.3 ng/mL for 15 or 30 mg and < 10 ng/mL for 60 mg, which corresponded to the 25th percentile in the HOKUSAI-VTE trial (Verhamme et al. 2016). Concentration-to-dose ratio (C/D-ratio) was calculated and compared between dose groups, as a dose-standardized measurement of exposure.

### Data sources

Data for this study was retrieved from two sources: the electronic health record system TakeCare and the analytics platform CGM Analytics (CompuGroup Medical). TakeCare is currently used within the Stockholm Region and contains comprehensive clinical and administrative data, including diagnoses, healthcare visits, treatments, and prescribed medications. CGM Analytics is a reporting platform that consolidates healthcare data, including laboratory results.

### Statistical methods

Our primary analysis was renal function in relation to edoxaban concentration. Secondary analyses included age and weight in relation to edoxaban concentration, presence of a pharmacokinetic drug-drug interaction, and off-label dosing. Descriptive data analysis was performed by calculating median (IQR), mean (standard deviation), 5–95th percentile and range. A *p*-value of < 0.05 was considered statistically significant. Wilcoxon rank sum exact test (Mann Whitney *U* test) and *t*-test were used for comparisons between groups. Spearman test was used to assess correlation between covariates and concentration. Statistical analysis was performed in Stata/BE 18.0.

## Results

### Study participants (patients)

**Fig. 1:**
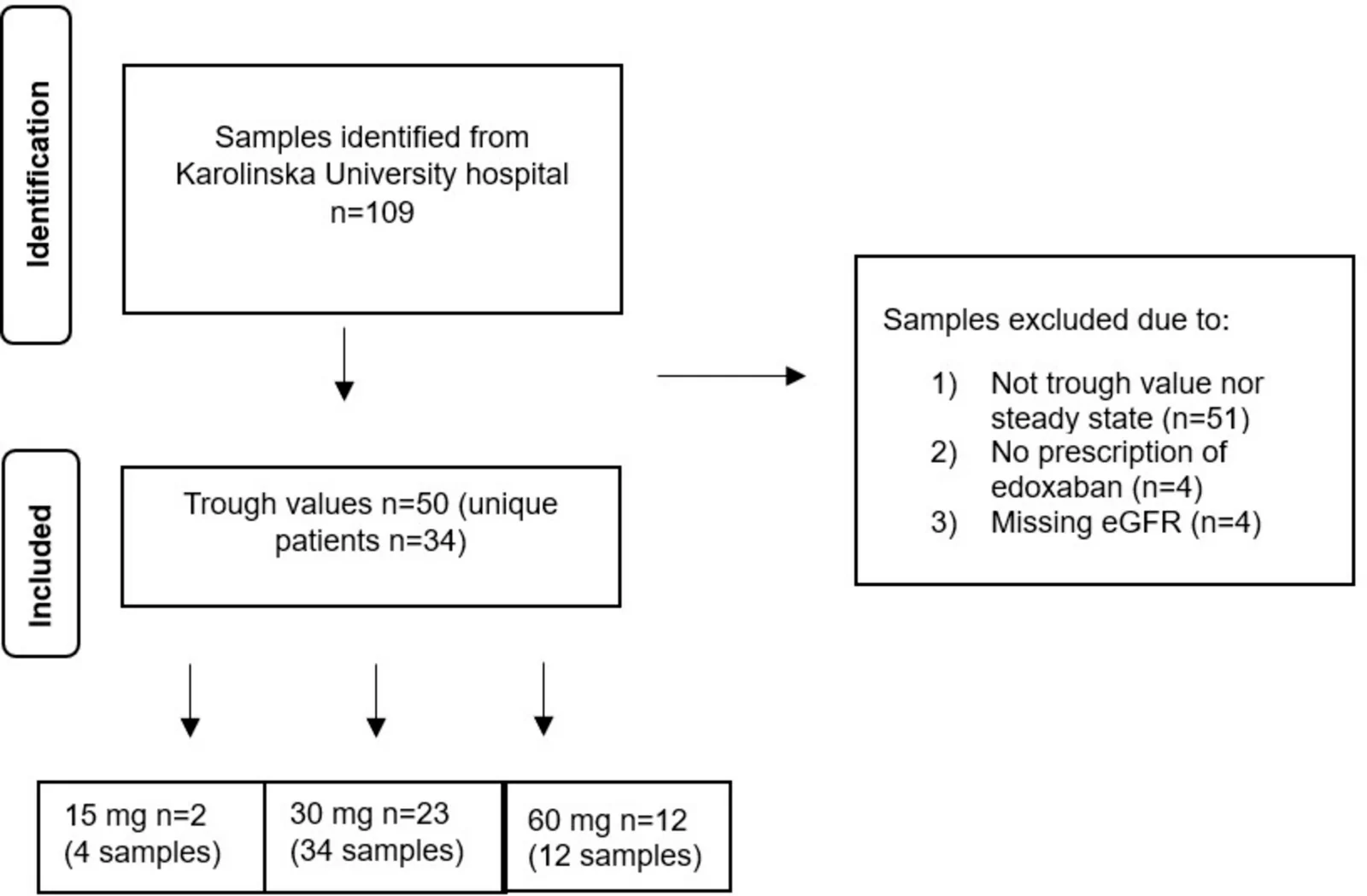
Flow chart of inclusion of edoxaban samples and patients. The study population consisted of 34 patients (50 trough edoxaban samples)

A total of 109 samples from 55 individuals analyzed for edoxaban concentration were identified. Out of these, 50 samples (34 patients) were determined to be trough values at steady state with complete data on eGFR and prescription of edoxaban and were included in the analysis (Fig. 1).

The majority of patients (62%) were treated with edoxaban 30 mg and had a registered diagnosis of VTE (65%) (Table 1). Patients treated with 60 mg compared to 30 mg edoxaban were younger (median 52.5 vs. 71 years, *p* < 0.05) and had higher eGFR (median 83 vs. 39 ml/min/1,73m^2^, *p* < 0.05 (Table 2).

**Table 1:** Patient characteristics at the time of sampling and analysis of edoxaban

Number of patients, *n*	34
Number of samples, *n*	50
Samples per patient, mean ± SD (range), *n*	1.5 ± 1.1 (1–5)
Age, median (range), years	67.5 (26–80)
Sex, female *n* (%)	19 (56%)
Dose 60 mg, number of patients (nr of samples)^a^	12 (12)
Dose 30 mg, number of patients (nr of samples)^a^	23 (34)
Dose 15 mg, number of patients (nr of samples)^a^	2 (4)
Weight, median (IQR) (kg)^b^	78.3 (20.9)
eGFR, median (IQR) (mL/min/1.73 m^2^)	47.5 (38)
Atrial fibrillation (AF), *n* (%)	8 (24%)
Venous thromboembolism (VTE), *n* (%)	22 (65%)
Both AF and VTE, *n* (%)	2 (6%)
No documented AF or VTE, *n* (%)	6 (18%)

^a^Two patients were treated with both 30 mg and 15 mg, and one patient with both 30 mg and 60 mg over time

^b^Data on weight was missing in four patients

**Table 2:** Patient characteristics and trough concentrations stratified by dose group (i.e., 30 mg daily and 60 mg daily). Data on 15 mg is not shown due to few individuals

	All doses	Dose 30 mg	Dose 60 mg	*p*-value
No. of patients (*n*)	34	23	12	-
No. of samples (*n*)	50	34	12	-
Age (years) median (IQR)	78.3 (21.0)	71.0 (16.0)	52.5 (21.5)	< 0.05
eGFR (mL/min/1.73 m^2^) median (IQR)	47.5 (39.0)	39.0 (35.0)	83.0 (19.0)	< 0.05
Weight mean (kg) (SD)	76.4 (14.9)	75.2 (14.7)	79.6 (18.0)	0.55
Time between last dose and sampling time:				
Mean (SD) Median (IQR) 5th-95th percentile (min–max)	24.0 (3.5) 24.9 (2.7) 16.0–28.3 (15–29.2)	24.4 (3.4) 25.1 (2.0) 16.0–28.3 (15.0–28.8)	22.4 (4.0) 23.2 (5.8) 16.0–29.2 (16.0–29.2)	
Trough concentration (ng/mL)				
Median (IQR) 5th–95th percentile (min–max) (samples)	17.5 (20.9) 3.3–53.3 (1.8–57.6)	17.5 (25.3) 4.0–57.0 (2.3–57.6)	13.1 (7.6) 1.8–26.5 (1.8–26.5)	0.25
Trough concentration (ng/mL) median (IQR) 5th-95th percentile (min–max) (patient level *n* = 33)	13.2 (17.8) 3.3–50.7 (1.8–57.3)	16 (22.5) 4.0–50.7 (3.7–57.3)	12.9 (13.7) 1.8–26.5 (1.8–26.5)	0.34
Concentration to dose ratio median (IQR)	0.4 (0.9)	0.6 (0.8)	0.2 (0.2)	< 0.05

### Edoxaban trough concentrations

Low edoxaban concentration (< 12 ng/mL in patients with a daily dose of 15 or 30 mg and < 16 ng/mL in patients with a daily dose of 60 mg, respectively) was found in 42% of samples (21/50). More precisely, low concentrations were observed in eight samples (67%) of patients treated with 60 mg, and in 13 samples (38%) of patients treated with 30 mg. Using the threshold from Hokusai-VTE trial, low edoxaban concentration was present in 15 out of 50 samples corresponding to 30% of the study population, similar to the expected count according to Hokusai VTE trial concentration data (Verhamme et al. 2016).

### Edoxaban plasma concentration in relation to dose and eGFR

**Fig. 2:**
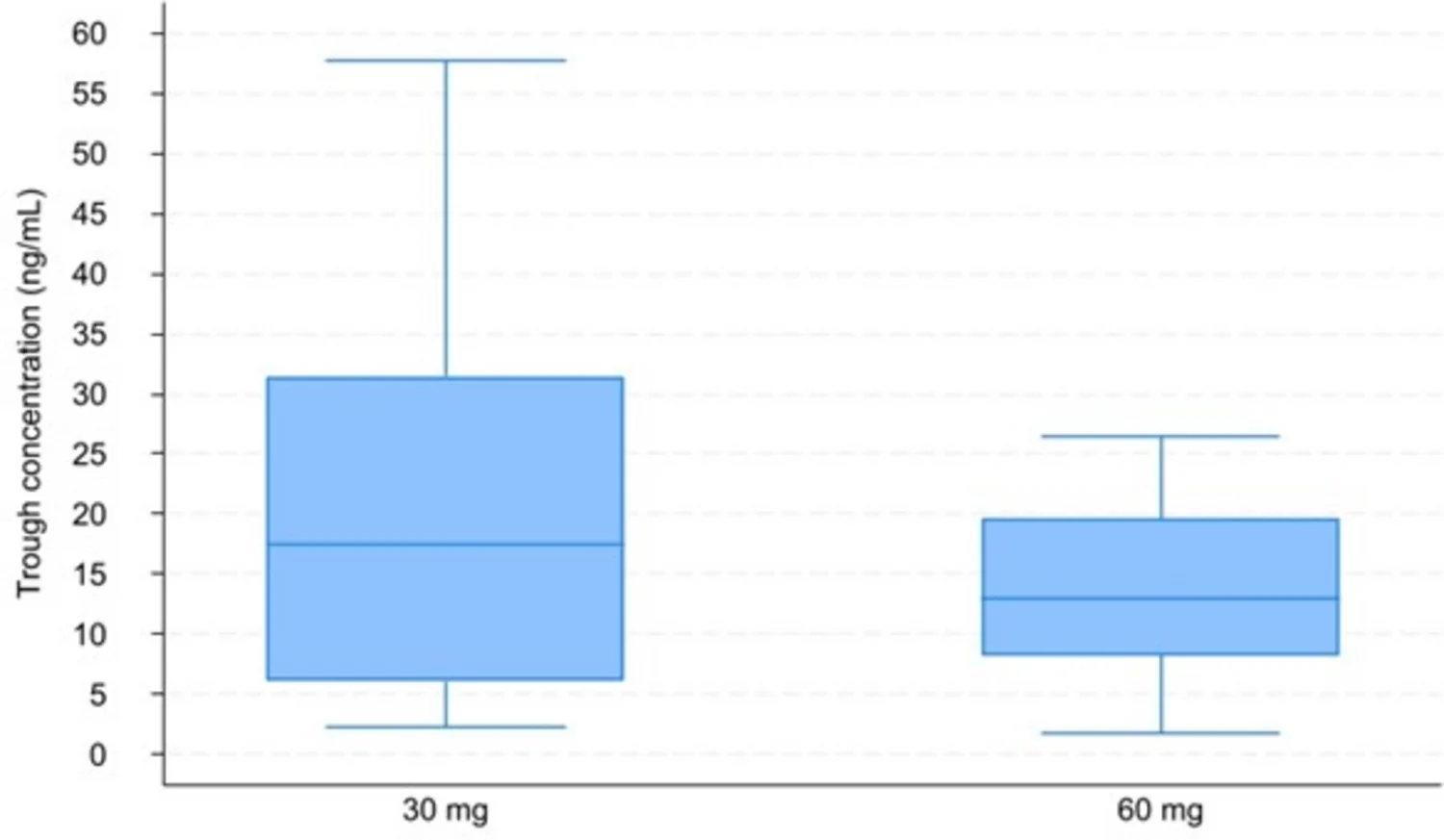
Boxplot showing the distribution of edoxaban trough concentrations (ng/mL) in 30 mg (*n* = 34) and 60 mg dose groups (*n* = 12) analyzed *per sample*. The central line represents the median concentration, while the box covers the interquartile range (IQR). The whiskers extend to the minimum and maximum values. There was no statistically significant difference in concentration between dose groups (*p* = 0.25)

There was no difference in edoxaban concentration in the samples of patients treated with 60 mg compared to 30 mg (Fig. 2, Table 2). However, concentration-to-dose ratio was three times higher in the 30-mg group compared to 60-mg group which implies relatively higher edoxaban concentration in the 30-mg group compared to 60-mg group (Table 2).

**Fig. 3:**
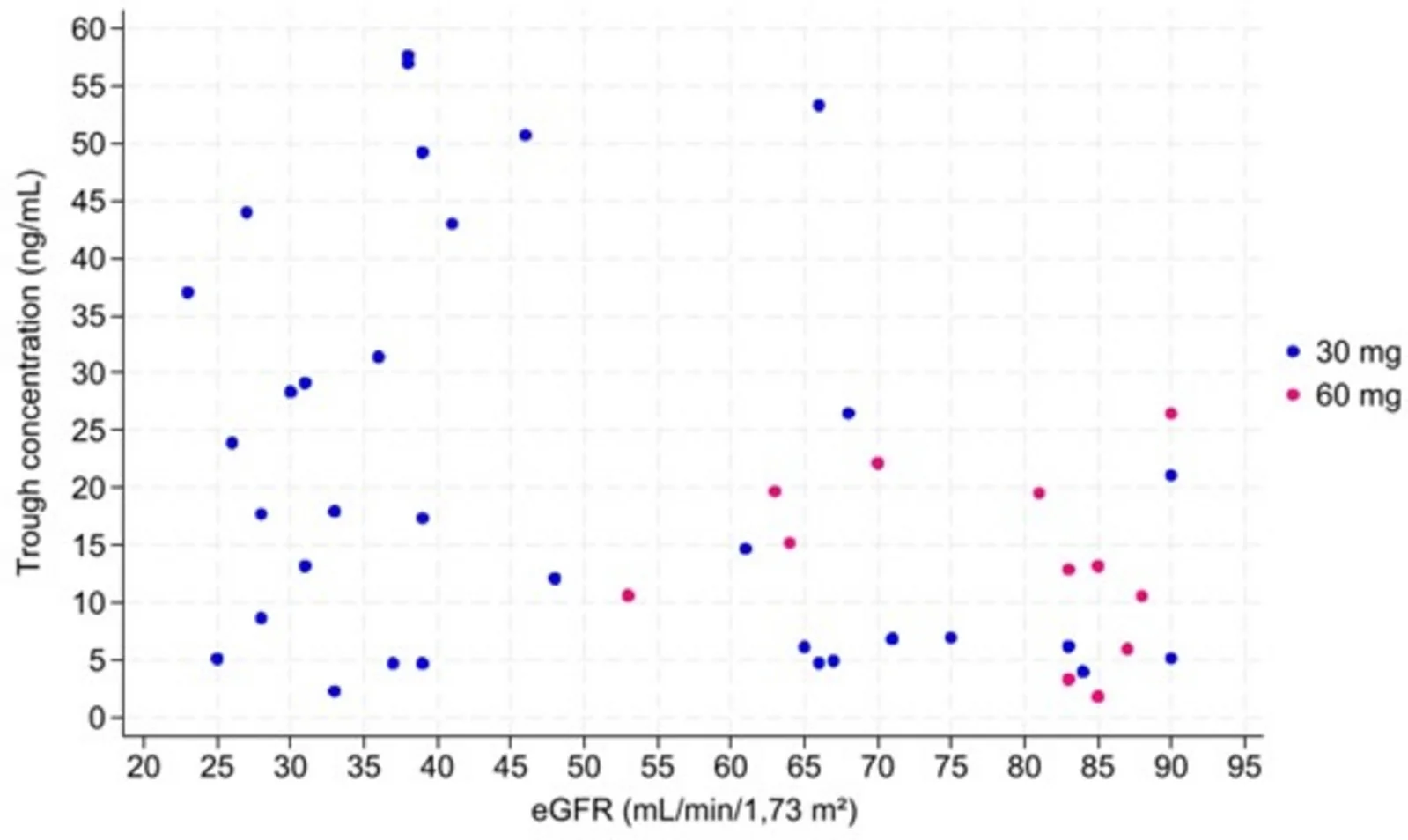
Scatter plot showing the relationship between eGFR (mL/min/1.73 m^2^) and trough edoxaban concentration in patients with edoxaban 30 mg daily (blue dot) and 60 mg daily (pink dot). Each dot represents one sample (*n* = 46)

A high interindividual variability of edoxaban concentrations was observed in patients treated with edoxaban 30 mg (range 2.3–57.6 ng/mL) (Table 2, Fig. 3, Figure S3, S5). Scatterplots are presented for both sample-level (Fig. 3) and patient-level analyses (Figure S3).

**Fig. 4:**
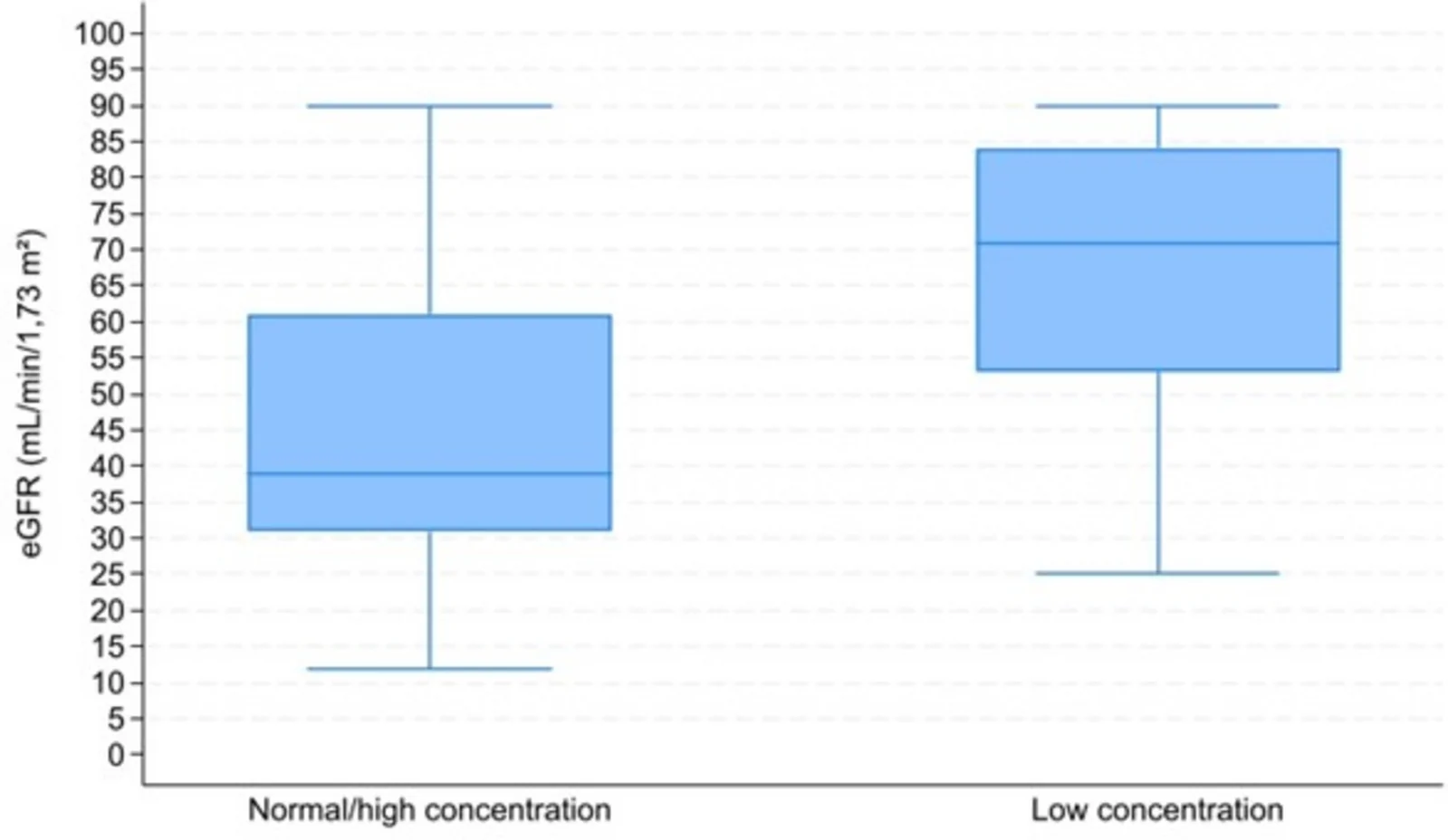
Boxplot showing estimated glomerular filtration rate (eGFR) in the group with low concentration (< 12 ng/mL in patients treated with 15 or 30 mg and < 16 ng/mL in patients with 60 mg) and normal or high concentrations. Higher eGFR was observed in the low-concentration group compared to normal/high concentrations (median eGFR 71 mL/min/1.73 m^2^ vs eGFR 39 mL/min/1.73 m^2^, *p* = 0.004). All samples and doses were included in the analysis (*n* = 50)

In the samples with low concentration, the eGFR was higher compared to normal/high concentrations (median eGFR 71 mL/min/1.73 m^2^ vs eGFR 39 mL/min/1.73 m^2^, *p* = 0.004) (Fig. 4). No significant difference in age and weight was observed in patients with low compared to normal/high concentrations. Other potential causes for low concentrations were off-label dose reductions (*n* = 4) and DDIs (*n* = 4). In ten patients, no identifiable cause for low concentrations was found.

The sensitivity analyses (edoxaban concentration in individual patients) showed similar distributions and patterns as for analyses of samples and statistical significance remained (Figure S2-S4).

When analyzing concentrations for all dosages in all samples (*n* = 50), a weak correlation was found between eGFR and concentration (Spearman’s rho =  − 0.4; *p* < 0.05) indicating that higher eGFR values were associated with lower drug concentrations (Fig. 3). After stratifying on dose, no correlation was observed in the 30-mg groups (Spearman’s rho =  − 0.3; *p* = 0.1) (Figure S5). No correlation was found between concentration and weight or between concentration and age.

### Potential drug-drug interactions

At least one potential pharmacokinetic drug-drug interaction was identified in 14 of 34 patients (41%). Drugs potentially decreasing the concentration of edoxaban and concomitantly prescribed to patients with low concentration included carbamazepine (*n* = 2), phenytoin (*n* = 1), and rifampicin (*n* = 1). Concomitant medication leading to increased concentration of edoxaban prescribed in patients with high concentration included tacrolimus (*n* = 1), ciclosporin (*n* = 2), and amiodarone (*n* = 1). Edoxaban concentration was high (range 43–73 ng/mL) in four patients concomitantly treated with P-gp inhibitors (tacrolimus, amiodarone, or ciclosporin) despite dose reduction to 30 mg, indicating lower clearance than expected.

## Discussion

This study provides valuable insights into the interindividual variability of edoxaban plasma concentrations and factors influencing concentration levels in a clinical setting. The strengths of the study include clinical data and consecutive samples of edoxaban concentrations in patients from a tertiary hospital analyzed in the same laboratory with a validated method for quantifying low levels of edoxaban.

Our results show lower than expected trough concentrations in approximately 40% of patients compared to the reference values from the ENGAGE AF-TIMI 48 trial, which included patients with atrial fibrillation. However, when using reference values from patients treated for VTE (Hokusai-VTE trial), the concentrations were not lower than expected. A higher renal function, measured as eGFR, was found in patients with low edoxaban concentration compared to patients with normal or high concentration.

Our study showed no statistically significant difference in plasma concentrations between dose groups. In contrast with our findings, lower concentrations were observed in patients treated with 30 mg compared to 60 mg in clinical trials and observational studies (Lin et al. 2021; Ruff et al. 2015; Steffel et al. 2021b; Verhamme et al. 2016). In the ENGAGE AF-TIMI trial, the concentration-to-dose ratio was similar in both dose groups (approximately 0.4), while the median edoxaban concentrations were lower in the group treated with 30 mg compared to the group treated with 60 mg (Steffel et al. 2021b). In comparison, the concentration-to-dose ratio in our study was three times higher in the 30-mg group compared to 60-mg group (0.6 vs. 0.2), which suggests relatively lower concentrations in the 60-mg group.

Renal function could be one possible contributing factor of the high interidividual variability of edoxaban concentrations. A weak correlation was found between eGFR and edoxaban concentration; however, these findings should be interpreted with caution due to the limited strength of the association. A relationship between renal function and edoxaban exposure is both plausible and expected, given the pharmacokinetic properties of edoxaban, and has been shown in previous studies (Clapham et al. 2024; Lin et al. 2021; Ono et al. 2022). Edoxaban undergoes 50% renal elimination, while metabolism to active metabolites (< 10%) and biliary excretion accounts for the remaining elimination (“SmPC edoxaban” n.d.). In patients with atrial fibrillation and high renal clearance, the SmPC recommends a careful assessment due to the potential risk for reduced effectiveness in patients with creatinine clearance > 95 mL/min (Bohula et al. 2016; “SmPC edoxaban” n.d.).

Our results showed that age and weight were not significantly associated with low concentrations. Other studies have shown that body weight influences edoxaban concentrations, although unaffected exposure in patients with weight up to 140 kg was observed in a recent study (Clapham et al. 2024). Edoxaban is not recommended in patients with BMI > 40 kg/m^2^ due to lack of data (Martin et al. 2021). The lack of association with weight in our study should be interpreted with caution as BMI was not available for our cohort and due to lack of data on weight in some patients.

DDIs with antiepileptics or rifampicin could be an explanation for a few of the low concentrations in our study. Co-prescription of drugs with potential for drug-drug interactions seem to be a common indication for TDM since 41% of our study population had concomitant treatment with drugs that could either increase or decrease edoxaban concentration. Data on dietary supplements was not available but could be a potential cause of low concentration of edoxaban, since, for example, St John’s Wort (hypericum perforatum) can interact with edoxaban by inducing P-gp and/or cytochrome P450 3A4 (CYP3A4).

Some patients in our study had off-label dose reductions, which also has been reported in previous observational studies, ranging from 26% (Kirchhof et al. 2024) to 33% (Lin et al. 2021). Off-label dose reduction has been associated with higher rates of all-cause and cardiovascular mortality compared to regular dosing; however, this could be secondary to off-label dose reduction in frailer and older populations (Kirchhof et al. 2024). These findings highlight the importance of addressing and avoiding off-label underdosing in clinical practice. In such cases, TDM can be of value since it can show whether there is a need to increase the dose.

Given that our cohort included patients with both VTE and AF, as well as a subset without clearly documented indications, defining a single expected target range for plasma concentrations is challenging. This raises the possibility that applying AF-based thresholds to a mixed population with VTE and AF may lead to misclassification, with some VTE patients categorized as having “low” concentrations despite having levels consistent with those observed in VTE trials.

The cutoffs of low vs. normal/high concentrations were defined using the 5th percentile of edoxaban levels as reported in the ENGAGE AF-TIMI 48 trial (Ruff et al. 2015; Steffel et al. 2021b). In Hokusai VTE trial (Verhamme et al. 2016), the trough concentrations were lower compared to ENGAGE AF-TIMI 48 trial. Differences in trough concentrations between trials may reflect differences in patient characteristics, such as age and renal function. This choicewas motivated by the large sample size of this trial and the availability of detailed percentile distributions of edoxaban concentrations. However, this approach has important limitations, as ENGAGE AF-TIMI 48 included a population with AF, whereas our cohort predominantly consisted of patients with VTE and fewer patients with AF.

Having a trough concentration below the 5th percentile of a reference population does not necessarily imply subtherapeutic exposure or increased clinical risk of cardiovascular events. The relationship between edoxaban plasma concentrations and clinical outcomes remains incompletely elucidated and therapeutic ranges have not been firmly established for routine clinical use. Therefore, the categorization of concentrations as “low” in this study should be interpreted with caution and primarily as a relative measure rather than a definitive indicator of underexposure.

There are currently evidence-based thresholds of expected trough concentrations, but evidence-based guidance regarding dose adjustments is lacking. However, according to practical guidelines, expected edoxaban trough concentration has a range of 12–43 ng/mL (Steffel et al. 2021a, b). In clinical practice, patients with edoxaban trough concentration below 12 ng/mL would probably be classified as “low.” In our study, two patients receiving 30 mg edoxaban daily had high plasma concentrations and were subsequently dose reduced to 15 mg, suggesting that TDM could serve as a supportive tool in individualized treatment decisions. However, these observations should be interpreted with caution, as they are based on a small sample size and are therefore uncertain. Further research in larger cohorts is needed to define clinically relevant concentration thresholds.

### Limitations

The study is limited by its small sample size, which reduces statistical power and increases the risk of type II errors. Additionally, small and unstable estimates could introduce greater uncertainty which could contribute to an increased risk of type I errors. The results should be interpreted as hypothesis generating, and additional studies are needed to confirm these observations.

The observational study design introduces potential bias, particularly selection bias related to the reasons for referral for TDM. Edoxaban plasma level measurement is not routinely performed in all patients, but rather in specific clinical situations such as therapeutic failure, bleeding, potential drug-drug interactions or renal failure. The study patients and samples included in this study are therefore probably not representative of the general edoxaban-treated population. The high proportion of low concentrations observed in this study might partly reflect this selection and limit the generalizability of the results. However, it offers valuable information on factors potentially affecting plasma concentrations of edoxaban in a clinical setting where edoxaban is measured to further individualize treatment.

The active metabolites of edoxaban have similar pharmacological activity as edoxaban but are not measured in our laboratory method. Hence, a low concentration of edoxaban could possibly still be therapeutic considering a potential increased concentration of active metabolites (Siriez et al. 2020). However, the clinical trials did not present results of the metabolite levels (Ruff et al. 2015; Verhamme et al. 2016).

We did not have access to patients’ BMI which would have further improved the precision of estimated renal function; however, the usage of LM-rev formula is an advantage over Chronic Kidney Disease Epidemiology Collaboration (CKD-EPI) and Modification of Diet in Renal Disease (MDRD) Formulas in the Swedish population for estimating renal function (Björk et al. 2020, SBU Report 2012). Retrieving information from electronic health records can result in incomplete or inaccurate data which introduces uncertainty about adherence and should be considered when interpreting the findings. The inclusion of samples collected 15–30 h after the last dose was a pragmatic decision, based on the half-life of edoxaban (10–15 h), to allow for variability in sampling. However, this wide sampling window may have introduced variability of trough concentration measurements.

## Conclusion

Therapeutic drug monitoring of edoxaban plasma concentrations from a tertiary center revealed a higher proportion of low edoxaban plasma concentrations at a higher proportion than expected. Patients with low edoxaban concentrations were more likely to have normal renal function. Additionally, off-label dose reductions and potential drug-drug interactions were observed in some cases. The small sample size and the selected study population limit the generalizability of the findings. However, the results suggest a potential role for therapeutic drug monitoring in selected patients to support individualized treatment. Our report should be regarded as hypothesis generating, and larger studies are needed to confirm these observations.

## Supplementary Information

Below is the link to the electronic supplementary material.

ESM 1 (DOCX 194 KB)

## Data Availability

The datasets generated during the current study are not publicly available due to privacy restrictions but are available from the corresponding author on request.

## Declarations

### Clinical trial registration

The study was registered in the Region Stockholm Clinical Studies Database prior to study initiation.

### Plain English summary

In this retrospective study of patients with measured plasma concentrations of the anticoagulant edoxaban, 42% of samples showed concentrations below expected thresholds. No significant difference between concentrations was observed between patients with 30 mg and 60 mg dosing. Patients with low edoxaban concentration were more likely to have normal renal function. Additionally, off-label dose reductions and drug-drug interactions were observed in some cases with low concentrations. These findings suggest a potential role of therapeutic drug monitoring in selected patients to support individualized treatment, although larger studies are needed to confirm these observations.
