# Supplementary material for: Low plasma edoxaban concentrations during therapeutic drug monitoring: a single-center retrospective study
Source: Naunyn Schmiedebergs Arch Pharmacol. 2026 Jun 3;399(11):17899–908. doi: 10.1007/s00210-026-05486-4 (PMC13503419; doi:10.1007/s00210-026-05486-4)
Supplement: Supplementary file 1 [file 210_2026_5486_MOESM1_ESM.docx]

**SUPPLEMENTARY MATERIAL**

Low plasma edoxaban concentrations during therapeutic drug monitoring-
A single-centre retrospective study

Paulina Flis, Roza Chaireti, Rickard E. Malmström, Karolina Nowinski

**Table S1.** Definition of drugs with potential for pharmacokinetic interaction with edoxaban. Only prescribed medications ≤1 month before or at sampling date were included.

| **Dronedarone** | C01BD07 |
| --- | --- |
| **Amiodarone** | C01BD01 |
| **Verapamil** | C08DA01 |
| **Diltiazem** | C08DB01 |
| **Quinidine** | C01BA01 |
| **Clarithromycin** | J01FA09 |
| **Erythromycin** | J01FA01 |
| **Itraconazole** | J02AC02 |
| **Ketoconazole** | H02CA03 |
| **Posaconazole** | J02AC04 |
| **Ticagrelor** | B01AC24 |
| **Carbamazepine** | N03AF01 |
| **Apalutamide** | L02BB05 |
| **Enzalutamide** | L02BB04 |
| **Mitotane** | L01XX23 |
| **Phenobarbital** | N03AA02 |
| **Phenytoin** | N03AB02 |
| **Primidone** | N03AA03 |
| **Rifampicin** | J04AB02 |
| **Rifamycin** | J04AB03 |
| **Imatinib** | L01EA01 |
| **Crizotinib** | L01ED01 |
| **Vandetanib** | L01EX04 |
| **Sunitinib** | L01EX01 |
| **Abiraterone** | L02BX03 |
| **Cyclosporin** | L04AD01 |
| **Tacrolimus** | L04AD02 |
| **Oxcarbazepine** | N03AF02 |
| **Levetiracetam** | N03AX14 |
| **Valproate** | N03AG01 |
| **Azithromycin** | J01FA10 |
| **Atazanavir** | J05AE08 |
| **Lopinavir/Ritonavir** | J05AR10 |
| **Ritonavir** | J05AE03 |
| **Nirmatrelvir / Ritonavir** | J05AE30 |
| **Darunavir / Cobicistat** | J05AR14 |
| **Darunavir** | J05AE10 |
| **Lapatinib** | L01EH01 |
| **Telithromycin** | J01FA15 |
| **Voriconazole** | J02AC03 |
| **Lonafarnib** | A16AX20 |
| **Fosamprenavir** | J05AE07 |
| **Tipranavir** | J05AE09 |

**Table S2:** ICD-10 diagnoses used for establishing indication for treatment with edoxaban.

**Diagnoses registered last 12 months before sampling:**

ICD I26: Pulmonary Embolism

ICD I80: Phlebitis and Thrombophlebitis

ICD I81: Splenic Vein Thrombosis

ICD I82: Other Venous Embolism and Thrombosis

ICD Z86.7A: Personal History of Venous Thrombosis

ICD Z86.7B: Personal History of Pulmonary Embolism

**Diagnoses registered at any timepoint before sampling:**

ICD I48: Atrial fibrillation or flutter

**SUPPLEMENTARY FIGURES:**

**Figure S2.** Boxplot showing the distribution of edoxaban trough concentrations (ng/mL) in 30 mg (n= 22) and 60 mg dose groups (n= 11) analysed *per patient*. In patients with multiple samples, the median concentration of the samples per patient calculated. One patient was excluded from this analysis due to both 30 mg and 60 mg dosing. Two patients were treated with 15 mg and 30 mg, and in this analysis only the median concentrations at 30 mg dose was included. There was no statistically significant difference in concentration between dose groups (p=0.34).

**Figure S3**: Scatter plot showing the relationship between median eGFR (mL/min/1.73 m²) and median trough edoxaban concentration (ng/mL) in patients with edoxaban 30 mg daily (blue dot) and 60 mg daily (pink dot). One patient with both 30 mg and 60 mg dosing was excluded from this analysis. Each dot represents one patient (n=33).

**Figure S4**: Boxplot showing distribution of median eGFR in the group with low median concentration and normal/high median concentrations (cut-off for low concentration <12 ng/mL in patients treated with 30 mg and <16 ng/mL in patients with 60 mg). Analysis was performed per patient and dose (n=33). Patients with 15 mg dose were not included in this analysis. One patient was excluded from the analysis due to both 30 mg and 60 mg dosing. Higher eGFR was observed in the low median concentration group compared to normal/high median concentrations (median eGFR 69 mL/min/1.73 m² vs eGFR 39 mL/min/1.73 m², p = 0.03).

**Figure S5:** Scatter plot showing the relationship between eGFR (mL/min/1.73 m²) and trough edoxaban concentration (ng/mL) following a 30 mg dose (n = 34). A substantial interindividual variability of concentrations can be observed. Each dot represents one sample.
